# Supplementary material for: Genome-wide identification of Apetala2 gene family in Hypericum perforatum L and expression profiles in response to different abiotic and hormonal treatments
Source: PeerJ. 2023 Aug 28;11:e15883. doi: 10.7717/peerj.15883 (PMC10470449; doi:10.7717/peerj.15883)
Supplement: Supplemental Information 1 [file peerj-11-15883-s001.docx]

**Table S1.** Ks, Ka, and Ka/Ks ratios of the eight gene pairs.

| Gene pairs | Ka value | Ks value | Ka/Ks ratio |
| --- | --- | --- | --- |
| *HPAP2_1*&*HPAP2_18* | 1.0150 | 0.9436 | 1.0757 |
| *HPAP2_2*&*HPAP2_9* | 5.2324 | 1.7027 | 3.0730 |
| *HPAP2_3*&*HPAP2_13* | 0.2209 | 0.2903 | 0.7608 |
| *HPAP2_4*&*HPAP2_6* | 0.9844 | 1.0534 | 0.9345 |
| *HPAP2_7*&*HPAP2_21* | 0.0016 | 0.0229 | 0.0683 |
| *HPAP2_8*&*HPAP2_12* | 0.0050 | 0.0382 | 0.1319 |
| *HPAP2_14*&*HPAP2_16* | 1.0140 | 0.9471 | 1.0707 |
| *HPAP2_19*&*HPAP2_20* | 0.9831 | 1.0678 | 0.9208 |

**Table S2.** Primers used for real-time fluorescent quantitative PCR of stress response genes.

| Primer name | Primer sequences (5’-3’) |
| --- | --- |
| *HpAP2_5*-F | CGGGAAAGATGGAGCATTGT |
| *HpAP2_5*-R | AAACCCAAGAGCCTCCTGAC |
| *HpAP2_11*-F | GGCTTATGACAAAGCTGCGA |
| *HpAP2_11*-R | AAGCTCACCGGAAGACGTAA |
| *HpAP2_12*-F | GCCAGGACAAATGCCTCAAT |
| *HpAP2_12*-R | AAGCAAACCTGGTGACTCCT |
| *HpAP2_17*-F | TGGGTTGTTCGACAACGAAG |
| *HpAP2_17*-R | GAGGTTGCAACCTGAGGCAA |
| *HpAP2_18*-F | AGGAAGTCCATCGACACGTT |
| *HpAP2_18*-R | TGCAGCTGTTGTCCCATAGA |
| *ACT2*-F | AGGAGTCCCTCCACGACCAC |
| *ACT2*-R | GCCGTTGTGTACCGGGTAGG |
